# Real-world evidence of the use of the infliximab biosimilar SB2: data from the PERFUSE study

**DOI:** 10.1093/rap/rkad031

**Published:** 2023-04-17

**Authors:** Bruno Fautrel, Yoram Bouhnik, Philippe Dieude, Pascal Richette, Maxime Dougados, Ulrich Freudensprung, Amira Brigui, Janet Addison

**Affiliations:** Rheumatology Department, Pitié-Salpêtrière Hospital, Sorbonne University, AP-HP, Paris, France; Pierre Louis Institute for Epidemiology and Public Health, INSERM UMRS 1136, Paris, France; Paris IBD Center, Groupe Hospitalier Privé Ambroise Paré—Hartmann, Neuilly-sur-Seine, France; Department of Rheumatology, Paris-Cité University, AP-HP, Paris, France; Hôpital Bichat-Claude Bernard, INSERM UMR1152, Paris, France; Rheumatology Department, Hôpital Lariboisière, AP-HP, Paris, France; Department of Rheumatology, Paris-Cité University, Hôpital Cochin, AP-HP, Paris, France; Clinical Epidemiology and Biostatistics, INSERM (U1153): PRES Sorbonne Paris-Cité, Paris, France; Biogen GmbH, Baar, Switzerland; Biogen France SAS, Paris, France; Biogen IDEC, Maidenhead, UK

**Keywords:** SB2, biosimilar, infliximab, switch, rheumatoid arthritis, psoriatic arthritis, axial spondyloarthritis

## Abstract

**Objective:**

PERFUSE is a non-interventional study of 1233 adult patients (rheumatology, *n *=* *496; IBD, *n *=* *737) receiving routine infliximab (IFX) biosimilar SB2 therapy. The aim of this report was to investigate the 12-month persistence, effectiveness and safety outcomes of routine SB2 treatment in patients with chronic inflammatory rheumatic disease.

**Methods:**

Patients with a diagnosis of RA, PsA or axial spondyloarthritis (axSpA) were assigned to one of three study cohorts according to whether SB2 treatment initiated after September 2017 had been the first IFX treatment (IFX naïve) or followed transition from reference IFX (IFX ref) or another IFX biosimilar (IFX bs). Outcomes to month 12 (±2) included persistence (primary outcome), SB2 dose, disease status, immunogenicity and safety.

**Results:**

At month 12, persistence on SB2 in IFX-naïve, IFX ref and IFX bs cohorts, respectively, [mean percentage (95% CI)] by indication was as follows: 59% (36.1, 76.2), 75% (57.5, 86.1) and 85% (69.6, 93.0) for RA (*n* = 98); 64% (34.3, 83.3), 87% (65.6, 95.7) and 83% (60.0, 93.1) for PsA (*n* = 62); and 56% (44.4, 66.5), 80% (70.8, 86.1) and 80% (72.5, 85.6) for axSpA (*n* = 336). Disease activity was comparable at baseline and month 12 within the IFX ref and bs subgroups of all cohorts by indication. No immunogenicity concerns or new safety signals were detected.

**Conclusion:**

SB2 was safe and effective in IFX-naïve patients and in patients transitioned from prior IFX ref or bs.

**Trial registration:**

clinicaltrials.gov, NCT03662919

Key messages

- Data on real-world long-term use of the infliximab biosimilar SB2 in infliximab-naïve patients or those with prior infliximab treatment are sparse.
- PERFUSE is a long-term, non-interventional, multicentre study including patients with rheumatic diseases receiving SB2 as routine therapy.
- SB2 is safe and effective in infliximab-naïve patients and in those who transitioned from prior infliximab (reference or biosimilar); at 1 year post-initiation, most study patients were continuing on SB2 treatment.

## Introduction

SB2, a biosimilar of the reference anti-TNF-α antibody infliximab (IFX ref), received marketing authorization in the EU in May 2016 for use in all indications for which IFX ref is approved, including RA, Crohn’s disease, ulcerative colitis, radiographic axial spondyloarthritis (axSpA; also known as AS), PsA and psoriasis [1]. The marketing authorization of SB2 was based on demonstration of comparable physicochemical and biological characteristics [2], pharmacokinetic similarity in healthy patients [3] and comparable efficacy and safety in patients with RA compared with IFX ref [4].

A phase III study of participants with moderate to severe RA despite MTX treatment who were randomized to receive either IFX or SB2 showed comparable efficacy, safety and immunogenicity up to 54 and 78 weeks [4]. The regulatory approval process requires the conduct of randomized clinical trials in a highly controlled setting, on a selected patient population. However, physicians, health technology assessment bodies and reimbursement authorities welcome real-world evidence on a variety of outcomes in the routine clinical setting [5]. Real-world results on the long-term use of SB2 in patients who are either IFX naïve or who have received prior IFX ref or another IFX biosimilar (IFX bs) are sparse. The PERFUSE study addresses the need for such real-world evidence. Here, we describe the persistence, effectiveness, SB2 dose and safety outcomes of SB2 treatment in patients with rheumatology diagnoses, followed to 12 months post SB2 initiation.

## Methods

### Study design

PERFUSE (NCT03662919) is a long-term, non-interventional, multicentre study. The study was submitted to the Committee for the Protection of Persons (CPP) SUD-EST II and was approved on 21 March 2018 (ID-RCB).

Patients receiving SB2 as routine therapy, prescribed at physician discretion independently of study inclusion, were enrolled between June 2018 and July 2019 and were followed for 24 months at 21 specialist sites (12 gastroenterology and 9 rheumatology) across France. Findings from the rheumatology sites are reported in this article.

Adults aged ≥18 years with a physician-confirmed diagnosis of RA, PsA or axSpA and who were either IFX naïve or had received IFX ref or IFX bs prior to being initiated on SB2 [6] after September 2017 were enrolled into PERFUSE. (Other than SB2, the only other IFX biosimilar available for prescription during the study period was CT-P13.) Patients who were not expected to be followed up at the same rheumatology clinic for 2 years after SB2 initiation; patients with a primary diagnosis of psoriasis, rheumatoid juvenile arthritis, uveitis or hidradenitis suppurativa; and women of childbearing potential intending to become pregnant during study follow-up were excluded.

The switch was completely independent from study participation. The protocol was non-interventional and did not influence standard clinical practice. There were no protocol-specified assessments or procedures (including treatment adjustment).

Switch was not mandatory in France. Patients and/or clinicians were able to refuse switch/continue reference/receive any other therapies according to Institution/local practice and standard of care.

Clinical data were captured retrospectively and/or prospectively from patient records. Study visits coincided with routine hospital visits. Patients received written information about the study; informed consent was documented.

The database extract for this 12-month analysis was taken on 29 October 2020.

### Effectiveness and safety assessments

All data in PERFUSE were captured as part of routine clinical practice. As a non-interventional study, outcomes were measured according to the usual rhythm of patient visits, with flexibility at period milestones. For this analysis, outcomes were reported for three time points: baseline (time of SB2 initiation), and at month 6 (±2) and month 12 (±2) post initiation. The primary outcome measure of the study was SB2 treatment persistence from baseline to month 12. Patient characteristics at initiation of SB2 (age, gender, BMI, disease history and status, previous biologic treatments and treatments at the time of enrolment) were recorded, and outcomes related to the effectiveness, immunogenicity and safety of SB2 were assessed over 12 months.

Treatment effect (i.e. effectiveness) [7] was assessed via disease scores in routine use at each study site, including DAS28 [8, 9] for RA and PsA and BASDAI [10] for PsA and axSpA, and by disease status (high/low disease activity or remission). DAS28 remission is defined as a score ≤2.6. Low disease activity (LDA) is a score >2.6 to ≤3.2 according to DAS28 and a score <4 according to BASDAI. High disease activity (HDA) is a score >3.2 according to DAS28 and a score ≥4 according to BASDAI.

Safety outcomes include treatment-emergent adverse events (TEAEs) and serious adverse events (SAEs). All clinical and laboratory adverse events (AEs) were coded using Medical Dictionary of Regulatory Activities (MedDRA 24.1). Investigators specified the reasons for discontinuation of SB2. Immunogenicity data were collected only from centres that routinely gathered these data, and immunogenicity was determined based on the detection of serum anti-drug antibodies (ADAs) using the Lisa Tracker ELISA kits (Theradiag, Croissy-Beaubourg, France) [11].

### Statistical analysis

The ‘all enrolled patients’ population is defined as all eligible enrolled patients who had received at least one infusion of SB2 (i.e. infusion documented in the PERFUSE database). Baseline is defined as the date of SB2 initiation. Kaplan–Meier (KM) techniques were used to analyse the primary outcome measure (i.e. the proportion of patients who were still treated with SB2 at month 12). KM estimates of the quartiles (quartile 1, median, quartile 3), corresponding 95% CI and range (minimum, maximum) are presented.

Given that disease scores were assessed and captured only at baseline and study months 6 and 12, no imputation/replacement of missing values was performed. Continuous variables are reported as the mean, standard deviation, minimum, 25th centile (quartile 1), median, 75th centile (quartile 3), maximum and 95% two-sided CIs, where appropriate. Categorical variables are summarized as frequencies and percentages. Proportions are presented with 95% two-sided CIs. Disease remission and LDA are defined as a DAS28 score ≤2.6, and >2.6 to ≤3.2, respectively, and as a BASDAI score <4.0. SAS v.9.4 (Cary, NC, USA) was used in the statistical analysis.

## Results

### Patient disposition, demographics and baseline characteristics

PERFUSE (*n* = 1233) included 496 patients with inflammatory rheumatic disease (RA, *n* = 98; PsA, *n* = 62; axSpA, *n* = 336). Results from the final 12-month analysis are reported here. Clinical characteristics at baseline are presented in Table 1. The RA cohort was predominantly female, whereas the majority of patients in the PsA and axSpA cohorts were male. Concomitant medications taken by patients during the study period are listed in Supplementary Table S1, available at *Rheumatology Advances in Practice* online. Concomitant use of MTX was ∼67% in the RA cohort (mainly among patients with prior IFX use), 47% in the PsA cohort and 31% in the axSpA cohort.

**Table 1.** Clinical characteristics at baseline

Characteristic	*n*	RA cohort (*n* = 98)	*n*	PsA cohort (*n* = 62)	*n*	axSpA cohort (*n* = 336)
Age, mean (s.d.), years						
IFX naïve	22	53.1 (15.9)	14	48.5 (12.2)	81	43.1 (11.1)
Prior IFX ref	36	55.7 (13.5)	24	50.9 (10.0)	109	47.1 (12.4)
Prior IFX bs	40	58.8 (12.6)	24	55.3 (15.3)	146	50.2 (12.4)
Women, *n* (%)						
IFX naïve	22	16 (72.7)	14	6 (42.9)	81	26 (32.1)
Prior IFX ref	36	30 (83.3)	24	10 (41.7)	109	32 (29.4)
Prior IFX bs	40	30 (75.0)	24	5 (20.8)	146	47 (32.2)
Duration of disease, mean (s.d.), years						
IFX naïve	22	11.3 (9.8)	14	4.2 (3.9)	81	7.2 (9.3)
Prior IFX ref	36	21.3 (7.7)	24	11.8 (7.8)	109	15.3 (10.1)
Prior IFX bs	40	13.7 (8.3)	24	13.9 (14.1)	146	17.5 (12.6)
MTX, %						
IFX naïve	15	68.2	6	42.9	15	18.5
Prior IFX ref	22	61.1	10	41.7	27	24.8
Prior IFX bs	29	72.5	13	54.2	39	26.7

axSpA: axial spondyloarthritis; IFX: infliximab; IFX bs: infliximab biosimilar; IFX ref: reference infliximab.

### Persistence and reasons for discontinuation

**Figure 1.**
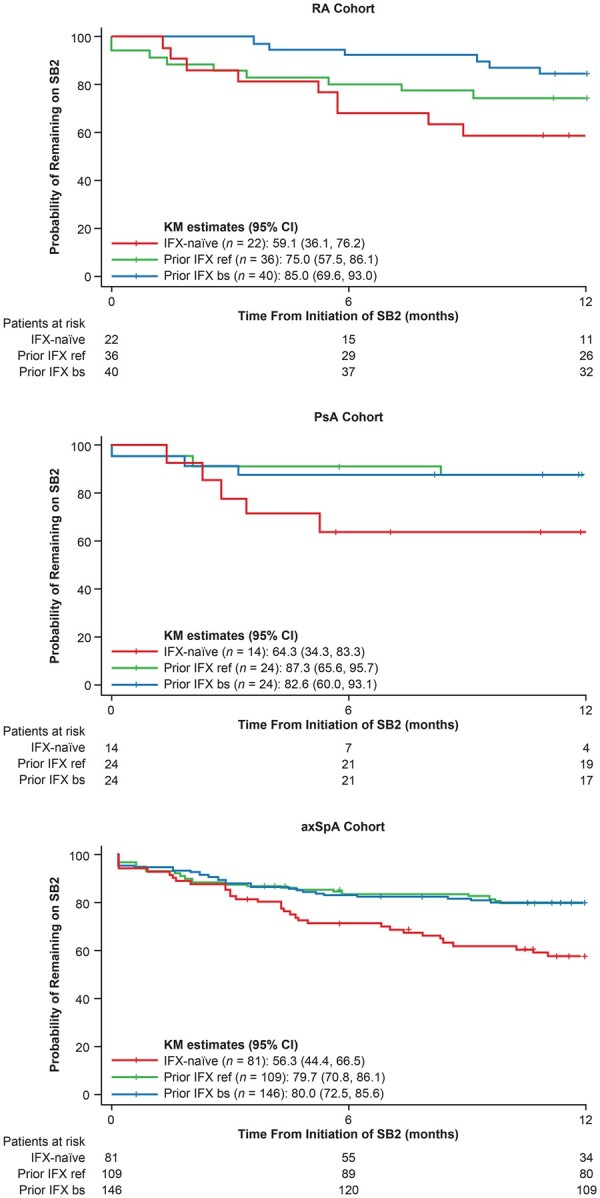
Persistence on SB2 over time. axSpA: axial spondyloarthritis; IFX: infliximab; IFX bs: infliximab biosimilar; IFX ref: reference infliximab; KM: Kaplan–Meier

At month 12, persistence (95% CI) on SB2 in IFX-naïve patients was as follows: RA, 59% (36.1, 76.2); PsA, 64% (34.3, 83.3); and axSpA, 56% (44.4, 66.5). Persistence in those with prior IFX ref was as follows: RA, 75% (57.5, 86.1); PsA, 87% (65.6, 95.7); and axSpA, 79.7% (70.8, 86.1). Persistence in patients with prior IFX bs was as follows: RA, 85.0% (69.6, 93.0); PsA, 82.6% (60.0, 93.1); and axSpA, 80.0% (72.5, 85.6). KM estimates (95% CI) of persistence on SB2 at month 12 are shown in Fig. 1.

The reasons for discontinuation are listed in Supplementary Table S2, available at *Rheumatology Advances in Practice* online. Discontinuations occurred most commonly among IFX-naïve patients (RA, 50%; PsA, 36%; axSpA, 52%). The most frequent reason for discontinuation was physician decision following loss of response. More than two-thirds of patients from the RA, PsA and axSpA cohorts received subsequent biological treatments after they had discontinued SB2 (Supplementary Table S3, available at *Rheumatology Advances in Practice* online).

### SB2 dose and disease activity evolution

**Figure 2.**
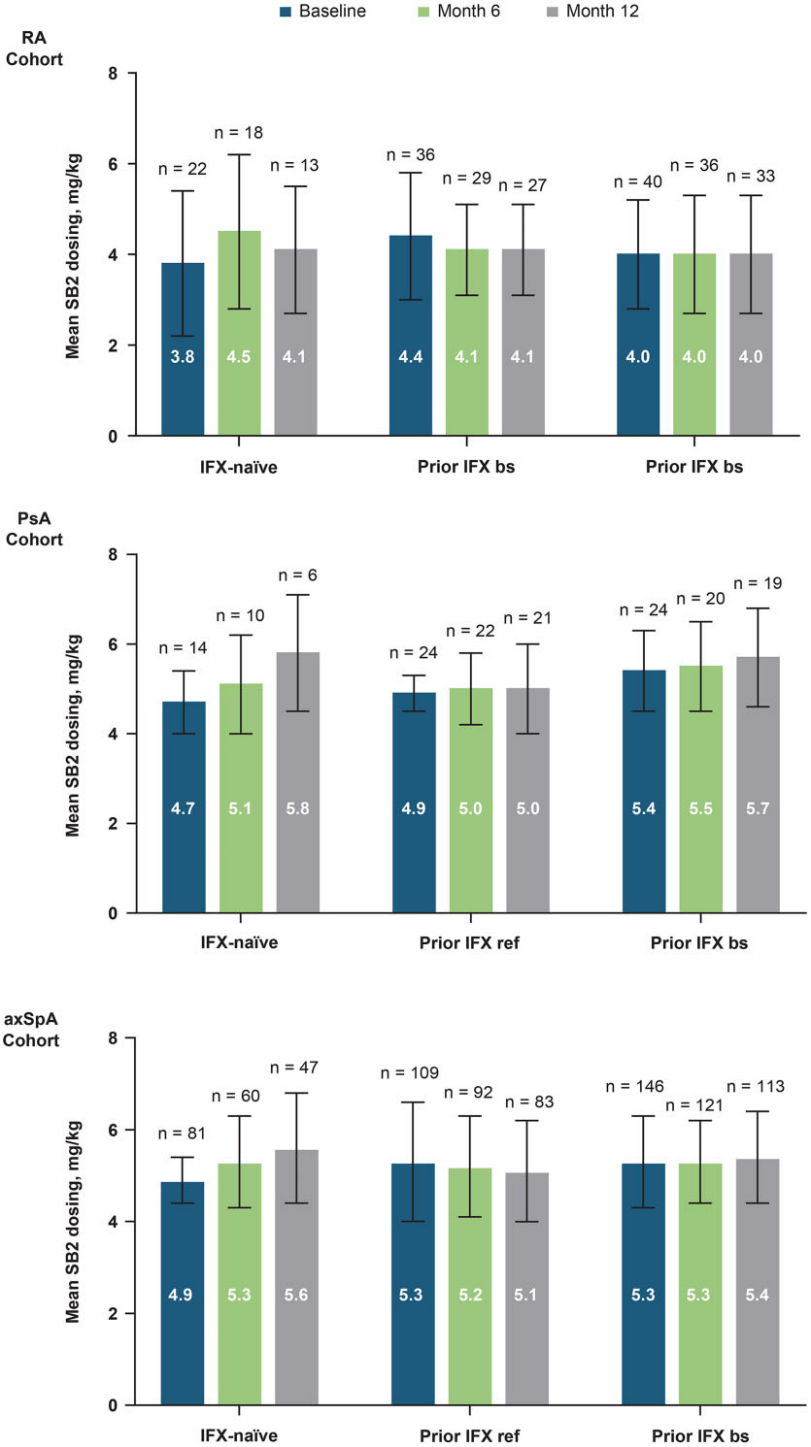
SB2 dose at baseline, month 6 and month 12. axSpA: axial spondyloarthritis; IFX: infliximab; IFX bs: infliximab biosimilar; IFX ref: reference infliximab

SB2 doses remained stable during the 12-month follow-up for the study in the RA, PsA and axSpA cohorts (Fig. 2; Supplementary Table S4, available at *Rheumatology Advances in Practice* online).

**Figure 3.**
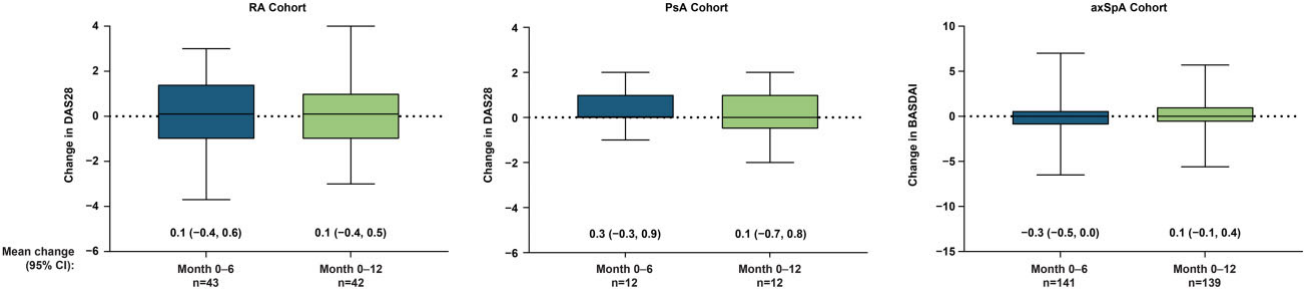
Change in disease score in patients transitioned from prior IFX^a^. ^a^Includes patients transitioned from either prior IFX ref or prior IFX bs. axSpA: axial spondyloarthritis; IFX bs: infliximab biosimilar; IFX ref: reference infliximab

Disease activity scores at baseline, month 6 and month 12 are shown in Table 2. The mean change in disease score from baseline to month 12 for the overall cohorts was as follows: RA, 0.0 (−0.4, 0.5; *n* = 48); PsA, 0.1 (−0.7, 0.8; *n* = 12); and axSpA, −0.1 (−0.5, 0.2; *n* = 162). In the IFX ref subgroups, mean changes (95% CI) in disease activity score from baseline to month 12 were 0.8 (0.2, 1.4) in RA (*n* = 15) and −0.1 (−0.9, 0.7) in PsA (*n* = 8) as measured by DAS28, and 0.0 (−0.5, 0.6) in axSpA (*n* = 52) as measured by BASDAI. In the IFX bs subgroups, mean changes (95% CI) in disease score from baseline to month 12 were −0.3 (−0.9, 0.3) in RA (*n* = 27), 0.4 (−1.9, 2.7) in PsA (*n* = 4) and 0.2 (−0.1, 0.5) in axSpA (*n* = 87). Fig. 3 shows the mean change in disease score from baseline to month 6 and from baseline to month 12 in patients pretreated with IFX ref or IFX bs in the three rheumatic cohorts. Apart from the prior IFX ref group in the RA cohort (69% in remission at baseline *vs* 47% in remission at month 12), the proportion of patients in remission at month 12 was either similar or higher than at baseline (Table 2).

**Table 2.** Disease scores and disease status

	RA cohort (*n*=98)			PsA cohort (*n*=62)			axSpA cohort (*n*=336)		
	IFX naïve (*n*=22)	Prior IFX ref (*n*=36)	Prior IFX bs (*n*=40)	IFX naïve (*n*=14)	Prior IFX ref (*n*=24)	Prior IFX bs (*n*=24)	IFX naïve (*n*=81)	Prior IFX ref (*n*=109)	Prior IFX bs (*n*=146)
Disease score, mean (95% CI); *n*									
DAS28-ESR									
Baseline	4.3 (2.7, 5.8); 8	2.3 (1.6, 3.0); 16	3.0 (2.5, 3.5); 19	2.0; 1	2.4 (0.8, 4.0); 8	1.7 (1.1, 2.2); 6			
Month 6	3.9 (2.9, 4.8); 8	2.9 (2.3, 3.5); 13	2.9 (2.4, 3.4); 18		1.8 (0.8, 2.9); 6	2.8 (1.2, 4.3); 4			
Month 12	2.5 (−16.6, 21.6); 2	2.8 (1.8, 3.8); 9	2.3 (1.9, 2.7); 17		1.8 (0.9, 2.6); 8	1.8 (1.0, 2.5); 4			
DAS28-CRP									
Baseline	4.7 (3.2, 6.1); 6	2.4 (1.3, 3.5); 10	2.6 (2.0, 3.2); 17		2.7 (−0.2, 5.5); 3	1.7 (−1.2, 4.5); 3			
Month 6	3.3 (1.9, 4.8); 3	2.4 (1.6, 3.3); 9	2.4 (1.3, 3.5); 9	3.5 (−2.9, 9.9); 2	2.7 (1.2, 4.1); 3	1.0; 1			
Month 12	3.4 (2.5, 4.3); 7	3.0 (2.2, 3.8); 10	2.8 (1.7, 3.9); 11	3.0; 1	2.3 (0.7, 3.8); 4	3.0 (0.5, 5.5); 3			
BASDAI									
Baseline							5.8 (5.3, 6.4); 54	3.2 (2.8, 3.7); 81	2.9 (2.5, 3.2); 125
Month 6							4.4 (3.6, 5.2); 42	2.9 (2.4, 3.4); 60	2.7 (2.2, 3.1); 99
Month 12							3.5 (2.5, 4.5); 30	2.8 (2.3, 3.4); 59	3.0 (2.6, 3.5); 92
Disease status, *n* (%)									
Baseline									
*n*	14	26	36	1	11	9	54	81	125
Remission	1 (7.1)^a^	18 (69.2)^a^	14 (38.9)^a^	1 (100)^a^	7 (63.6)^a^	8 (88.9)^a^	–	–	–
LDA	2 (14.3)^b^	4 (15.4)^b^	5 (13.9)^b^	0 (0)^b^	1 (9.1)^b^	0 (0)^b^	13 (24.1)^d^	49 (60.5)^d^	90 (72.0)^d^
MDA	6 (42.9)^c^	2 (7.7)^c^	16 (44.4)^c^	0 (0.0)^c^	2 (18.2)^c^	1 (11.1)^c^	–	–	–
HDA	5 (35.7)^d^	2 (7.7)^d^	1 (2.8)^d^	0 (0.0)^d^	1 (9.1%)^d^	0 (0.0)^d^	41 (75.9)	32 (39.5)	35 (28.0)
Month 6									
*n*	11	22	27	2	9	5	42	60	99
Remission	1 (9.1)^a^	10 (45.5)^a^	11 (40.7)^a^	0 (0.0)^a^	6 (66.7)^a^	3 (60.0)^a^	–	–	–
LDA	1 (9.1)^b^	5 (22.7)^b^	7 (25.9)^b^	0 (0.0)^b^	1 (11.1)^b^	1 (20.0)^b^	22 (52.4)^e^	42 (70.0)^e^	78 (78.8)^e^
MDA	8 (72.7)^c^	7 (31.8)^c^	8 (29.6)^c^	2 (100)^c^	2 (22.2)^c^	1 (20.0)^c^	–	–	–
HDA	1 (9.1)^d^	0 (0.0)^d^	1 (3.7)^d^	–	–	–	20 (47.6)^f^	18 (30.0)^f^	21 (21.2)^f^
Month 12									
*n*	9	19	28	1	12	7	30	59	92
Remission	2 (22.0)^a^	9 (47.4)^a^	17 (60.7)^a^	0 (0)^a^	9 (75.0)^a^	5 (71.4)^a^	–	–	–
LDA	0 (0.0)^b^	1 (5.3)^b^	3 (10.7)^b^	–	–	–	18 (60.0)^e^	43 (72.9)^e^	65 (70.7)^e^
MDA	6 (66.7)^c^	8 (42.1)^c^	6 (21.4)^c^	1 (100)^c^	3 (25.0)^c^	2 (28.6)^c^	–	–	–
HAD	1 (11.1)^d^	1 (5.3)^d^	2 (7.1)^d^	–	–	–	12 (40.0)^f^	16 (27.1)^f^	27 (29.3)^f^

For DAS28 ESR disease score and disease status in RA and PsA patients: ^a^remission: DAS28 ≤ 2.6; ^b^LDA (in RA, PsA): DAS28 > 2.6, ≤3.2; ^c^MDA (in RA, PsA): DAS28 > 3.2, ≤5.1; and ^d^HDA (in RA, PsA): DAS28 > 5.1.

For DAS28 CRP disease score in RA and PsA patients: remission: DAS28 ≤ 2.4.; LDA (in RA, PsA): DAS28 > 2.4, ≤2.9.; HDA (in RA, PsA): DAS28 > 4.64; MDA (in RA, PsA): DAS28 > 2.9, ≤4.64.

For BASDAI disease status in patients with axSpA: ^e^LDA (in axSpA): BASDAI <4.0; ^f^HDA (in axSpA): BASDAI ≥4.0.

axSpA: axial spondyloarthritis; HAD: high disease activity; ESR disease activity score 28; IFX: infliximab; IFX bs: infliximab biosimilar; IFX ref: reference infliximab; LDA: low disease activity; MDA: moderate disease activity.

CRP levels were comparable at baseline and month 12, respectively, in all prior IFX patients, regardless of diagnosis [median CRP, in milligrams per decilitre: RA, 3.2 (*n* = 49) and 3.2 (*n* = 54); PsA, 3.2 (*n* = 26) and 2.6 (*n* = 35); and axSpA, 3.0 (*n* = 178) and 2.0 (*n* = 173)]. Reductions in CRP levels were greatest among IFX-naïve patients [median CRP, in milligrams per decilitre: RA, 7.3 (*n* = 14) to 1.6 (*n* = 12); PsA, 17.9 (*n* = 10) to 5.4 (*n* = 6); and axSpA, 5.6 (*n* = 45) to 2.3 (*n* = 39)] (Supplementary Table S5, available at *Rheumatology Advances in Practice* online).

### Immunogenicity

No IFX-naïve patients in the RA (*n* = 22), PsA (*n* = 14) and axSpA (*n* = 81) cohorts had a history of ADA test either at baseline or during the study follow-up period (Table 3).

**Table 3.** Immunogenicity: antidrug antibody status at baseline and post-baseline

Patients	RA cohort			PsA cohort			axSpA cohort		
	ADA-positive test at baseline	ADA-negative at baseline	No history of ADA test at baseline	ADA-positive test at baseline	ADA-negative at baseline	No history of ADA test at baseline	ADA-positive test at baseline	ADA-negative at baseline	No history of ADA test at baseline
IFX-naïve patients, *n*	0	0	22	0	0	14	0	0	81
ADA-positive post-baseline^a^, *n*	0	0	1	0	0	1	0	0	6
ADA-negative post-baseline^b^, *n*	0	0	4	0	0	4	0	0	23
No post-baseline ADA measurement, *n*	0	0	17	0	0	9	0	0	52
Patients previously treated with IFX, *n*	0	0	76	0	1	47	2	8	245
ADA-positive post-baseline^a^, *n*	0	0	5	0	0	3	2	1	12
ADA-negative post-baseline^b^, *n*	0	0	12	0	1	17	0	5	48
No post-baseline ADA measurement, *n*	0	0	59	0	0	27	0	2	185

a At least one positive result during study follow-up.

b No positive result at any time during study follow-up.

ADA: antidrug antibody; axSpA: axial spondyloarthritis; IFX: infliximab.

In patients who were previously treated with IFX, none of the 76 RA patients, one of 47 PsA patients and 10 of 245 axSpA patients had a history of ADA testing at baseline, of whom 2 axSpA patients had at least one ADA-positive result. Both axSpA patients who were ADA positive at baseline maintained ADA positivity post-baseline; one of eight axSpA patients who tested ADA negative at baseline was ADA positive post-baseline.

Post-baseline ADA results were available for 17 RA patients, 21 PsA patients and 68 axSpA patients, of whom 5 RA patients, 3 PsA patients and 15 axSpA patients reported at least one ADA-positive test.

### Safety

Non-serious, related TEAEs for the IFX-naïve, prior IFX ref and prior IFX bs groups were reported as a proportion of each cohort as follows: RA, 13.6%, 11.1% and 12.5%; PsA, 0%, 8.3% and 4.2%; and axSpA, 14.8%, 15.6% and 11.6%, respectively. In total, 23 SAEs were reported for 20 patients. Two treatment-related SAEs were reported for two patients, both in the axSpA cohort (Supplementary Table S6, available at *Rheumatology Advances in Practice* online): hepatic cytolysis was reported for one IFX-naïve patient, and uveitis for one patient previously treated with IFX bs. Twenty-one unrelated SAEs were reported for 18 patients (Supplementary Table S7, available at *Rheumatology Advances in Practice* online).

## Discussion

The final month 12 data from the PERFUSE study rheumatology cohorts show no clinically meaningful differences observed in clinical effectiveness over a 12-month period in patients switched from IFX ref or IFX bs to SB2. More than 75% of patients who transitioned from prior IFX and continued on SB2 treatment to month 12 post SB2 initiation showed no meaningful difference in clinical measures, regardless of whether they received prior IFX ref or IFX bs. More than 56% of IFX-naïve patients continued on SB2 treatment at month 12 with no changes in dosing regimen.

Discontinuation rates of IFX bs have been reported to be higher in open-label studies but not in blinded studies [12], suggesting that knowledge of a switch to a biosimilar might affect patient perception and subsequent outcome. This perception is known as the nocebo effect, which is linked to anxious reactions to therapeutic interventions that occur because of negative expectations of the patient [13]. In our study, the persistence rate was found to be in line with the reported persistence rate for the biosimilar CT-P13 [14] and with the discontinuation rate reported for patients initiated on IFX ref [15], suggesting that although a nocebo effect cannot be ruled out, this did not have a meaningful impact in the population studied. It has been suggested that the patient–healthcare provider relationship is a key driver of acceptance of biosimilars and that prescriber and patient education might reduce the impact of the nocebo effect [16]. In PERFUSE, the most frequent reason for discontinuation was physician decision following loss of response.

The presence of ADA has been associated with a decrease in trough serum drug levels, lower clinical response and higher AE occurrence [17, 18]. In order to be approved as a biosimilar, meaningful differences in immunogenicity need to be excluded. However, there are still concerns in the minds of some prescribers about switching to biosimilars [19]. Therefore, it is important to assess immunogenicity data in the real-world setting. Although routine immunogenicity testing was infrequent in our study population, our results do not indicate an increased risk of immunogenicity to IFX following SB2 initiation.

Clinical and real-world studies, such as PERFUSE, provide evidence that patients can be switched effectively and safely from a reference drug to a biosimilar. For SB2, to date, there is one published clinical trial that investigated switching from IFX to SB2 in patients with RA [20], in which patients receiving IFX ref for the first 54 weeks were re-randomized to receive either IFX ref (IFX/IFX) or SB2 (IFX/SB2) for an additional 24 weeks. Patients receiving SB2 in the main study did not switch treatments (SB2/SB2). The efficacy, safety and immunogenicity profiles were reported to be comparable between the three treatment groups up to week 78, with no clinically meaningful immunogenicity after switching from IFX to SB2.

Another study evaluated the development of immunogenicity in 265 patients with chronic inflammatory diseases (RA, PsA, axSpA and IBD) observed over 3 years [21]. These patients were on maintenance therapy with IFX ref and successively switched to CT-P13 and then to SB2; IFX-naïve patients switched from a first to a second IFX bs (CT-P13 to SB2). The authors reported no increased risk of immunogenicity as a result of a single switch or successive switches to IFX bs, which corroborate our findings so far in PERFUSE [12, 14].

The PERFUSE study has limitations related to variabilities in clinical practices in the real-world setting, including missing data. Data in Table 2 were based on a small sample size. When missing data points were queried, some sites confirmed that disease scores are not calculated. In clinics in France, it is not mandatory to report disease scores. In addition, DASs by indication were as reported from the clinical practices. Although the AS DAS has been shown to have a better discriminatory capacity and sensitivity to change than BASDAI [22], the clinics included in this study routinely use BASDAI disease activity scores. Another limitation is that there were no control groups of patients continuing on reference or other IFX bs, because all eligible patients had been transitioned to SB2. The SB2 cohort could therefore not be compared with the IFX ref or IFX bs in terms of effectiveness, immunogenicity or safety.

In conclusion, these findings indicate that patients with RA, PsA or axSpA can be initiated successfully on SB2 as the first IFX therapy or can be transitioned effectively from prior IFX ref or IFX bs to SB2 without loss of response, with no dose penalty and with no safety concerns over 12 months. The use of biologic treatments has transformed the management of chronic inflammatory diseases. The introduction of biosimilar products in clinical practice can potentially reduce health expenditure [23]. A recent systematic review of 15 international studies found that non-medical switch from reference biologics to biosimilars (including IFX) resulted in a wide range of cost savings (about €7 to €13 739 per patient per year) [24, 25], reflecting market differences in regulatory and reimbursement systems [26]. Long-term studies and real-world studies using SB2 for the treatment of patients with chronic inflammatory rheumatic diseases will increase our understanding of the clinical utility of biosimilars and their potential to pave the way for increased access to treatment.

## Supplementary Material

rkad031_Supplementary_Data

## Data Availability

All data are included in the manuscript and supplementary materials.
