# Supplementary material for: Real-world evidence of the use of the infliximab biosimilar SB2: data from the PERFUSE study
Source: Rheumatol Adv Pract. 2023 Apr 17;7(2):rkad031. doi: 10.1093/rap/rkad031 (PMC10130189; doi:10.1093/rap/rkad031)
Supplement: rkad031_Supplementary_Data [file rkad031_supplementary_data.pdf]

SUPPLEMENTARY MATERIAL

Real-world evidence of the use of the infliximab biosimilar SB2 data from the PERFUSE study

Bruno Fautrel[1], Yoram Bouhnik[2], Philippe Dieude[3], Pascal Richette[4], Maxime Dougados[5], Ulrich Freudensprung[6], Amira Brigui[7], Janet Addison[8], on behalf of the PERFUSE investigators

[1]Rheumatology Department, Sorbonne University – AP-HP, Paris, and INSERM UMRS 1136, Pierre Louis Institute for Epidemiology and Public Health, Paris, France, [2]Beaujon Hospital, AP-HP, Paris-Cité University, Clichy, France, [3] Paris-Cité University, INSERM UMR1152, Paris, and Hôpital Bichat-Claude Bernard, AP-HP, Paris, France, [4]Hôpital Lariboisière, Paris, France, [5]Department of Rheumatology, Paris-Cité University, Hôpital Cochin, AP-HP, Paris, and INSERM (U1153): Clinical epidemiology and biostatistics, PRES Sorbonne Paris-Cité, Paris, France, [6]Biogen GmbH, Baar, Switzerland, [7]Biogen France SAS, Paris, France, and [8]Biogen IDEC, Maidenhead, UK.

SUPPLEMENTARY TABLE S1 Concomitant medications

	RA cohort (*N* = 98)			PsA cohort (*N* = 62)			axSpA cohort (*N* = 336)		
	IFX naïve	Prior IFX ref	Prior IFX bs	IFX naïve	Prior IFX ref	Prior IFX bs	IFX naïve	Prior IFX ref	Prior IFX bs
Patients enrolled and treated with SB2, *n*	22	36	40	14	24	24	81	109	146
Patients with concomitant medication, *n* (%)	18 (18.8)	22 (61.1)	33 (82.5)	8 (57.1)	10 (41.7)	13 (54.2)	25 (30.9)	30 (27.5)	50 (34.2)
Concomitant medication, *n* (%)									
Methotrexate	15 (68.2)	22 (61.1)	29 (72.5)	6 (42.9)	10 (41.7)	13 (54.2)	15 (18.5)	27 (24.8)	39 (26.7)
Prednisone	11 (50.0)	2 (5.6)	16 (40.0)	5 (35.7)	2 (8.3)	6 (25.0)	9 (11.1)	5 (4.6)	17 (11.6)
Leflunomide	1 (4.5)	–	2 (5.0)	1 (7.1)	–	–	2 (2.5)	–	–
Hydroxychloroquine	3 (13.6)	1 (2.8)	–	2 (14.3)	–	–	1 (1.2)	–	–
Sulfasalazine	–	1 (2.8)	1 (2.5)	–	–	–	1 (1.2)	2 (1.8)	–
Prednisolone	–	1 (2.8)	–	2 (14.3)	–	–	2 (2.5)	1 (0.9)	2 (1.4)
Methylprednisolone	–	–	–	–	–	–	1 (1.2)	–	–

axSpA: axial spondyloarthritis; IFX: infliximab; IFX bs: infliximab biosimilar; IFX ref: reference infliximab; PsA: psoriatic arthritis; RA: rheumatoid arthritis.

SUPPLEMENTARY TABLE S2 Reasons for discontinuation of SB2[a]

	RA cohort (*N* = 98)			PsA cohort (*N* = 62)			axSpA cohort (*N* = 336)		
	**IFX naïve (*n* = 22)**	**Prior IFX ref (*n* = 36)**	**Prior IFX bs (*n* = 40)**	**IFX naïve (*n* = 14)**	**Prior IFX ref (*n* = 24)**	**Prior IFX bs (*n* = 24)**	**IFX naïve (*n* = 81)**	**Prior IFX ref (*n* = 109)**	**Prior IFX bs (*n* = 146)**
Patients who discontinued SB2, *n* (%)	11 (50.0)	11 (30.6)	6 (15.0)	5 (35.7)	5 (20.8)	5 (20.8)	42 (51.9)	26 (23.9)	36 (24.7)
Reasons for discontinuation, *n* (%)									
Patient decision	3 (13.6)	1 (2.8)	0 (0.0)	1 (7.1)	1 (4.2)	3 (12.5)	5 (6.2)	4 (3.7)	2 (1.4)
Physician decision, primary loss of response	0 (0.0)	0 (0.0)	0 (0.0)	2 (14.3)	0 (0.0)	0 (0.0)	8 (9.9)	1 (0.9)	1 (0.7)
Physician decision, secondary loss of response	5 (22.7)	7 (19.4)	2 (5.0)	2 (14.3)	2 (8.3)	1 (4.2)	17 (21.0)	13 (11.9)	20 (13.7)
Adverse event	3 (13.6)	2 (5.6)	0 (0.0)	0 (0.0)	1 (4.2)	0 (0.0)	9 (11.1)	8 (7.3)	8 (5.5)
Sustained remission	0 (0.0)	0 (0.0)	1 (2.5)	0 (0.0)	0 (0.0)	1 (4.2)	0 (0.0)	0 (0.0)	0 (0.0)
Other reason	0 (0.0)	1 (2.8)	3 (7.5)	0 (0.0)	1 (4.2)	0 (0.0)	3 (3.7)	0 (0.0)	5 (3.4)

[a]Not all discontinuations had a reason for discontinuation specified.
axSpA: axial spondyloarthritis; IFX: infliximab; IFX bs: infliximab biosimilar; IFX ref: reference infliximab; PsA: psoriatic arthritis; RA: rheumatoid arthritis.

SUPPLEMENTARY TABLE S3 Subsequent biological treatments

	RA cohort (*N* = 98)			PsA cohort (*N* = 62)			axSpA cohort (*N* = 336)		
	**IFX naïve (*n* = 22)**	**Prior IFX ref (*n* = 36)**	**Prior IFX bs (*n* = 40)**	**IFX naïve (*n* = 22)**	**Prior IFX ref (*n* = 36)**	**Prior IFX bs (*n* = 40)**	**IFX naïve (*n* = 22)**	**Prior IFX ref (*n* = 36)**	**Prior IFX bs (*n* = 40)**
Patients who withdrew SB2, *n* (%)	11 (50.0)	11 (30.6)	6 (15.0)	5 (35.7)	5 (20.8)	5 (20.8)	42 (51.9)	26 (23.9)	36 (24.7)
Patients who took subsequent biological treatment, *n* (%)	8 (72.7)	10 (90.9)	2 (33.3)	3 (60.0)	5 (100)	3 (60.0)	27 (64.3)	24 (92.3)	28 (77.8)
Subsequent treatment									
Abatacept	2 (25.0)	1 (10.0)	0	–	–	–	–	–	–
Adalimumab	1 (12.5)	0	0 (0.0)	0	0	1 (33.3)	6 (22.2)	3 (13.0)	6 (21.4)
Certolizumab	0	1 (10.0)	0	1 (33.3)	0	0	1 (3.7)	2 (8.3)	1 (3.6)
Etanercept	0	0	1 (50.0)	1 (33.3)	0	0	1 (3.7)	2 (8.3)	1 (3.6)
Golimumab	0	0	1 (50.0)	0	1 (20.0)	0	3 (11.1)	1 (4.2)	1 (3.6)
Infliximab	0	7 (70.0)	0	0	2 (40.0)	2 (66.7)	5 (18.5)	10 (41.7)	13 (46.4)
Rituximab	1 (12.5)	0	0	–	–	–	–	–	–
Sarilumab	1 (12.5)	0	0	–	–	–	–	–	–
Secukinumab	–	–	–	0	1 (20.0)	0	10 (37.0)	3 (12.5)	4 (14.3)
Tocilizumab	3 (37.5)	0	0	–	–	–	0	0	1 (3.6)
Tofacitinib	0	1 (10.0)	0	–	–	–	0	1 (4.2)	0
Ustekinumab	–	–	–	1 (33.3)	0	0	0	1 (4.2)	0
Other	–	–	–	0	1 (20.0)	0	1 (3.7)	1 (4.2)	1 (3.6)

axSpA: axial spondyloarthritis; IFX: infliximab; IFX bs: infliximab biosimilar; IFX ref: reference infliximab; PsA: psoriatic arthritis; RA: rheumatoid arthritis.

Supplementary Table S4 SB2 dose (mg/kg) at baseline and month 12

	RA cohort (*N* = 98)			PsA cohort (*N* = 62)			axSpA cohort (*N* = 336)		
	n	**Mean (S.D.)**	**Q1, Q3**	***n***	**Mean (S.D.)**	**Q1, Q3**	***n***	**Mean (S.D.)**	**Q1, Q3**
IFX naïve									
Baseline	22	3.8 (1.6)	3.0, 5.0	14	4.7 (0.7)	5.0, 5.0	81	4.9 (0.5)	5.0, 5.0
Month 12	13	4.1 (1.4)	3.0, 5.0	6	5.8 (1.3)	5.0, 7.5	47	5.6 (1.2)	5.0, 7.0
Prior IFX ref									
Baseline	36	4.4 (1.4)	3.0, 5.0	24	4.9 (0.4)	5.0, 5.0	109	5.3 (1.3)	5.0, 5.0
Month 12	27	4.1 (1.0)	3.0, 5.0	21	5.0 (1.0)	5.0, 5.0	83	5.1 (1.1)	5.0, 5.0
Prior IFX bs									
Baseline	40	4.0 (1.2)	3.0, 5.0	24	5.4 (0.9)	5.0, 5.0	146	5.3 (1.0)	5.0, 5.0
Month 12	33	4.0 (1.3)	3.0, 5.0	19	5.7 (1.1)	5.0, 6.5	113	5.4 (1.0)	5.0, 5.0

axSpA: axial spondyloarthritis; IFX: infliximab; IFX bs: infliximab biosimilar; IFX ref: reference infliximab; PsA: psoriatic arthritis; Q1: quartile 1; Q3: quartile 3; RA: rheumatoid arthritis; S.D.: standard deviation.

Supplementary Table S5 CRP at baseline and month 12

	RA cohort ($N = 98$)			PsA cohort ($N = 62$)			axSpA cohort ($N = 336$)		
	n	**Median**	**IQR**	***n***	**Median**	**IQR**	***n***	**Median**	**IQR**
Baseline CRP, mg/L									
IFX naïve	14	7.3	1.3–11.0	10	17.9	8.0–27.8	45	5.6	2.0–16.0
Prior IFX ref	22	3.2	0.7–9.2	16	3.7	1.0–11.5	76	3.0	1.3–8.1
Prior IFX bs	27	3.2	2.0–5.9	10	3.2	1.0–7.2	102	2.3	0.9–7.0
Month-12 CRP, mg/L									
IFX naïve	12	1.6	0.0–3.8	6	5.4	2.3–17.0	39	2.3	0.9–10.3
Prior IFX ref	21	3.7	1.0–10.3	19	2.0	1.0–6.0	76	2.0	0.9–5.3
Prior IFX bs	33	3.0	1.0–4.9	16	3.4	2.1–6.9	97	2.1	1.0–5.0

axSpA: axial spondyloarthritis; CRP: C-reactive protein; IFX: infliximab; IFX bs: infliximab biosimilar; IFX ref: reference infliximab; IQR: interquartile range; PsA: psoriatic arthritis; RA: rheumatoid arthritis.

SUPPLEMENTARY TABLE S6 Related serious adverse events

	**RA cohort (*N* = 98)**			**PsA cohort (*N* = 62)**			**axSpA cohort (*N* = 336)**		
	**IFX naïve (*n* = 0)**	**Prior IFX ref (*n* = 0)**	**Prior IFX bs (*n* = 0)**	**IFX naïve (*n* = 0)**	**Prior IFX ref (*n* = 0)**	**Prior IFX bs (*n* = 0)**	**IFX naïve (*n* = 1)**	**Prior IFX ref (*n* = 0)**	**Prior IFX bs (*n* = 1)**
Eye disorders									
Uveitis	-	-	-	-	-	-	-	-	1
Hepatobiliary disorders									
Hepatic cytolysis	-	-	-	-	-	-	1	-	-

axSpA: axial spondyloarthritis; IFX: infliximab; IFX bs: infliximab biosimilar; IFX ref: reference infliximab; PsA: psoriatic arthritis; RA: rheumatoid arthritis.

SUPPLEMENTARY TABLE S7 Serious adverse events not related to treatment

	RA cohort (*N* = 98)			PsA cohort (*N* = 62)			axSpA cohort (*N* = 336)		
	**IFX naïve (*n* = 0)**	**Prior IFX ref (*n* = 0)**	**Prior IFX bs (*n* = 4)**	**IFX naïve (*n* = 0)**	**Prior IFX ref (*n* = 1)**	**Prior IFX bs (*n* = 1)**	**IFX naïve (*n* = 3)**	**Prior IFX ref (*n* = 3)**	**Prior IFX bs (*n* = 9)**
Cardiac disorders									
Acute heart failure	-	-	-	-	-	-	-	1	-
Gastrointestinal disorders									
Colitis	-	-	-	-	-	-	-	-	1
Sciatic hernia	-	-	1	-	-	-	-	-	-
General disorders and administration-site conditions									
Systemic inflammatory response syndrome	-	-	-	-	1	-	-	-	-
Infections and infestations									
Anal abscess	-	-	-	-	-	-	1	-	-
Pneumonia/Lung infection	-	-	-	-	-	-	-	-	1
Injury, poisoning and procedural complications									
Alcohol poisoning	-	-	-	-	-	-	1	-	-
Ankle fracture	-	-	-	-	-	-	-	-	1
Fall	-	-	-	-	-	-	-	-	1
Tibia fracture	-	-	-	-	-	-	-	-	1
Investigations									
Microbiology test abnormal	-	-	-	-	-	-	-	1	-
Musculoskeletal and connective tissue disorders									
Arthritis/coxitis	-	-	-	-	-	-	-	1	-
Spinal stenosis	-	-	1	-	-	-	-	-	-
Neoplasms: benign, malignant and unspecified									
Breast cancer	-	-	1	-	-	-	-	-	-
Prostate cancer	-	-	1	-	-	-	-	-	-
Nervous system disorders									

Cervical radiculopathy	-	-	-	-	-	-	1	-	-
Respiratory, thoracic and mediastinal disorders									
Dyspnea	-	-	-	-	-	-	-	-	1
Epistaxis	-	-	-	-	-	-	-	-	1
Skin and subcutaneous tissue disorders									
Dyshidrosis/dyshidrotic eczema	-	-	-	-	-	-	-	-	1
Surgical/medical procedures									
Knee arthroplasty	-	-	-	-	-	1	-	-	-
Renal surgery	-	-	-	-	-	-	-	-	1

axSpA: axial spondyloarthritis; IFX: infliximab; IFX bs: infliximab biosimilar; IFX ref: reference infliximab; PsA: psoriatic arthritis; RA: rheumatoid arthritis.
